# LincRNA Cox-2 Regulates Lipopolysaccharide-Induced Inflammatory Response of Human Peritoneal Mesothelial Cells via Modulating miR-21/NF-*κ*B Axis

**DOI:** 10.1155/2019/8626703

**Published:** 2019-12-03

**Authors:** Yaoyao Bian, Lili Yang, Bin Zhang, Wen Li, Sen Wang, Shuling Jiang, Xi Chen, Wenlin Li, Li Zeng

**Affiliations:** ^1^School of Nursing, Nanjing University of Chinese Medicine, Nanjing, China; ^2^School of First Clinical Medicine, Nanjing University of Chinese Medicine, Nanjing, China; ^3^Jingwen Library, Nanjing University of Chinese Medicine, Nanjing, China; ^4^Digestive Department, Ningbo Hospital of traditional Chinese Medicine, Ningbo, China; ^5^School of Preclinical Medicine, Guiyang University of Chinese Medicine, Guiyang, China; ^6^Department of Anorectal, Huainan Second People's Hospital, Huainan, China

## Abstract

Postoperative peritoneal adhesion (PPA) is a common postoperative complication caused by any peritoneal inflammatory process. This study aimed to identify the biological function of large intergenic non-coding RNAs (lincRNAs) Cox-2 in the inflammation reaction of adhesion formation. The Cox-2 expression in peritoneal adhesion tissues and normal tissues was detected. The human peritoneal mesothelium cells (HPMCs) were treated with lipopolysaccharide (LPS) to induce inflammatory injury. The effect of Cox-2 suppression on cell viability, apoptosis and inflammatory factors of LPS induced HPMCs injury were explored. The regulatory correlation between Cox-2 and miR-21, as well as the targeted genes of miR-21 were identified. Meanwhile, the regulatory mechanism of Cox-2/miR-21 axis on NF-*κ*B pathway was explored. It indicated that Cox-2 was highly expressed in peritoneal adhesion tissues compared with that in normal tissues. Suppression of Cox-2 ameliorated LPS induced HMPCs injury as cell viability was promoted, and cell apoptosis and the production of inflammatory factors were inhibited. And suppression of Cox-2 reversed the LPS induced HPMCs injury by regulation of miR-21 negatively. miR-21 was negatively correlated with TLR4, and TLR4 was predicted as target gene of miR-21. Furthermore, the suppression of miR-21 on LPS induced HPMCs injury was reversed by knockdown of TLR4, which could inhibited the activation of NF-*κ*B pathway axis. It suggested that the effect of Cox-2 on LPS induced HPMCs injury was achieved by negatively regulation of miR-21 and targeted TLR4 through NF-*κ*B pathway axis. The findings may provide a new insight into preventing postoperative peritoneal adhesion.

## 1. Introduction

Postoperative peritoneal adhesion (PPA) is a common iatrogenic complication after abdominal surgery with high incidence rate of 90% to 95% [1]. It remains an urgent clinical concern either in traditional open surgery or in laparoscopic procedure due to the significant acute intestinal obstruction or necrosis, chronic adhesive symptoms, such as abdominal pain, female infertility [2]. Increasing studies focus on the etiology, pathogenesis, risk factors, as well as effective preventive interventions [3]. Several evidences showed that the inflammatory response as well as the followed immune response participate in the whole process of adhesion occurrence and formation [4, 5]. However, the underlying mechanisms has not been fully elucidated. Thus, it is urgent need to understand the potential mechanisms of adhesion formation in cellular and molecular levels.

High-throughput sequencing suggested that either long non-coding RNAs (lncRNAs) or short non-coding microRNAs (miRNAs) play pivotal roles in genetic transcription and epigenetic regulation [6, 7]. Mounting evidences indicated that both lncRNAs and miRNAs function as key contributors in the pathogenesis and progression of a wide variety of cellular processes, including immune response [8, 9], inflammatory response [10, 11]. However, knowledge of the genome scale of lncRNAs, miRNAs and their potential biological functions associated with PPA remains limited.

Large intergenic non-coding RNAs (lincRNAs) are a new group of lncRNAs function as key regulators of distinct cellular function. LincRNA cyclooxygenase-2 (Cox-2) plays as a key mediator in inflammatory response [9] and immune-regulatory activities [12]. And miR-21 is a well-studied in inflammatory disease via binding to receptors of the Toll-like receptor (TLR) family [13]. Here, we tried to explore the specific function and underlying mechanism of lincRNA Cox-2 and miR-21 on the inflammatory response induced by lipopolysaccharide (LPS) through NF-*κ*B pathway axis.

## 2. Materials and Methods

### 2.1. Human Tissue Collection

Based on previous study [14], the human adhesion tissues were harvested from patients who underwent laparotomy for adhesive intestinal obstruction. The normal tissues were obtained from patients who underwent laparotomy for non-adhesive intestinal obstruction. With the informed consent of patients and under the approval of Ethic Committee of Huainan Second People's Hospital, all tissues were frozen in liquid nitrogen and then stored in -80°C freezer.

### 2.2. Animals and Models Preparation

Sixty male SD rats weighting 200 ± 20 g were obtained from Shanghai Sippr-BK laboratory animal Co. Ltd. (Shanghai, China). The rats were housed at a temperature and light-controlled room (12-h light/darkness cycle) with plenty of food and water. The rats were allowed to acclimatize this condition 3 days before use and then were randomly divided into six groups, that is, control (n = 30), PPA_1_ (one day after surgery, n = 6), PPA_3_ (3 days after surgery, n = 6), PPA_5_ (5 days after surgery, n = 6), PPA_7_ (7 days after surgery, n = 6) and PPA_14_ (14 days after surgery, n = 6) groups. All animal experiment were approved by the Guidelines of Accommodation and Care for Animals formulated by the Chinese Convention for the protection of vertebrate animals used for experimental and other scientific purposes and were authorized by the Laboratory Animal Management Committee of the Nanjing University of Chinese Medicine. The adhesion models were prepared as previously described [15]. A 1.5–2 cm midline incision was made to access the abdominal cavity after anaesthetized. The cecum of rats were pulled out smoothly on the wet gauze and scraped by a file for 5 min until the spot bleeding appeared on the cecum. Then, the damaged cecum was replaced into the abdominal cavity and the abdominal wall was sutured. The experimental rats were sacrificed at 1, 3, 5, 7 and 14 days after surgery and the cecum were collected for the following analysis.

### 2.3. Cell Culture

The human peritoneal mesothelium cells (HPMCs HMrSV5) were obtained from Honsun Biological Technology Co., Ltd. (Shanghai, China). HPMCs were seeded in RPMI 1640 medium (Gibco, Thermo Fisher Scientific, USA) with 10% fetal bovine serum (Gibco, Thermo Fisher Scientific, USA), and 100 U/mL penicillin and 100 *μ*g/mL streptomycin in the incubator (Panasonic, Japan) under 5% CO_2_ at 37°C. The cells were treated by LPS (Solarbio) with three different concentration (4, 8 and 12 ng/mL) for 24 hours to induce peritoneal inflammatory response. The cells without LPS were treated as control group.

### 2.4. Infection of Lentivirus Vector

HPMCs were cultured at 2 × 10^5^ cells per well in 6-well plates. Then these cells were infected with lentiviral particles carrying sequences against human Cox-2 (sh-Cox-2; Santa Cruz Biotechnology, USA) and negative control sequence (sh-NC; Santa Cruz Biotechnology, USA) for 48 hours, respectively. Finally, qRT-PCR was applied to determine the infection efficiency.

### 2.5. Oligonucleotide Transfection

The complementary DNA (cDNA) encoding Cox-2 was PCR-amplified, which were ligated into the pcDNA3.1 vector to form pcDNA-Cox-2. Oligonucleotides of miR-21 mimic, mimic negative control (mimic NC), siRNA against TLR4 (si-TLR4) and siRNA against the negative controls (si-NC) were obtained from Biomics Biotechnology Co., Ltd. The miR-21 inhibitor and inhibitor negative control (inhibitor NC) were purchased from Invitrogen (USA). The oligonucleotides were then added into Lipofectamine 2000 (Thermo Fisher Scientific, USA) to transfect the HPMCs.

### 2.6. Cell Viability Assay

2 × 10^4^ HPMCs were seeded in 96-well plates. After synchronization, the cells were different treated. At the second day, 20 *μ*L MTT (Sigma-Aldrich, USA) was added into the cells. Then, the HPMCs were incubated for 4 hours at room temperature. The absorbance at 490 nm was measured by a microplate reader (Tecan Infinite F50, Switzerland).

### 2.7. Cell Apoptosis Assay

2 × 10^4^ HPMCs were seeded with different treatments for 24 hours in 96-well plates. The cells were harvested and stained with Annexin V-FITC and 7-AAD, which were purchased from BD Biosciences. The cells were detected with a flow cytometry within 1 hours. Then the Annexin V^+^ cells were analyzed with software.

### 2.8. Enzyme-Linked Immunosorbent Assay (ELISA)

After different treatments, the supernatant of HMPCs were collected. The levels of four inflammatory factors (TNF-a, IL-1*β*, IL-6, IL-8) were determined by ELISA kit (R&D, USA) according to manufacturer's protocols.

### 2.9. Target Prediction

Targetscan (available online: http://www.targetscan.org/vert_72/) was used to predict the possible binding sequences of miR-21. It is a comprehensive online tool of predicted miRNA-target interactions.

### 2.10. Luciferase Reporter Assay

2 × 10^5^ HPMCs were prepared in 24-well plates and co-transfected with mutant TLR4 (TLR4-mut) or non-mutant TLR4 (TLR4-wt), miR-21 mimic or mimic NC with Lipofectamine 2000 reagent (Invitrogen, USA) for 48 hours. The Dual-Luciferase Reporter Assay System (Promega, USA) were applied to measure the luciferase activity.

### 2.11. Western Blot Analysis

HPMCs with different treatments were harvested and total protein was extracted by RIPA buffer (CWBIO, China). After qualified by Qubit2.0 and fractioned by 10% SDS-PAGE, the proteins were transfected to PVDF membranes (Millipore, USA). The membranes were incubated with primary antibodies (Santa Cruz Biotechnology, USA) overnight at 4°C. After further incubation with second antibodies for 1.5 hours at 37°C, the band visualization were viewed by Chemiluminescence Imaging system (Bio-Rad, USA) and the targeted proteins were determined by ImageLab Software.

### 2.12. qRT-PCR

Total RNAs was extracted from HPMCs and adhesion tissues in accordance with the manufacturer's instructions of a Trizol reagent (Invitrogen, USA). All primers were designed and synthesized from GenScript. The relative expression levels were determined using comparative Ct method formula 2^−ΔΔCT^. GAPDH was served as reference control for lncRNAs and mRNAs, while U6 was served as internal control for miRNAs, respectively.

### 2.13. Imaging Flow Cytometry Analysis

1 × 10^6^ HPMCs were harvested and washed after different treatments. After fixation, incubation with primary antibody against p-NF-*κ*B p65 (Santa Cruz Biotechnology, USA) for 2 hours at 4°C, the cells were incubated FITC-conjugated secondary antibody (CWBIO, China) for 30 min at 37°C. The nuclei of HPMCs were stained by 7-AAD (Invitrogen USA) for 5 min. The cells were visualized under an imaging flow cytometry (Amnis, Milllipore, USA).

### 2.14. Statistical Analysis

All experiments were repeated in triplicate. Quantitative variables were presented as the mean ± standard deviation. All data were carried out by SPSS 19.0. A two tailed Student's t-test was used when statistical significance between two different groups. And standard ANOVA methodology was applied for multiple group comparison. Significant difference was set as *P*<0.05.

## 3. Results

### 3.1. High Expression of lincRNA Cox-2 in Human and Rat Adhesion Tissues

**Figure 1:**
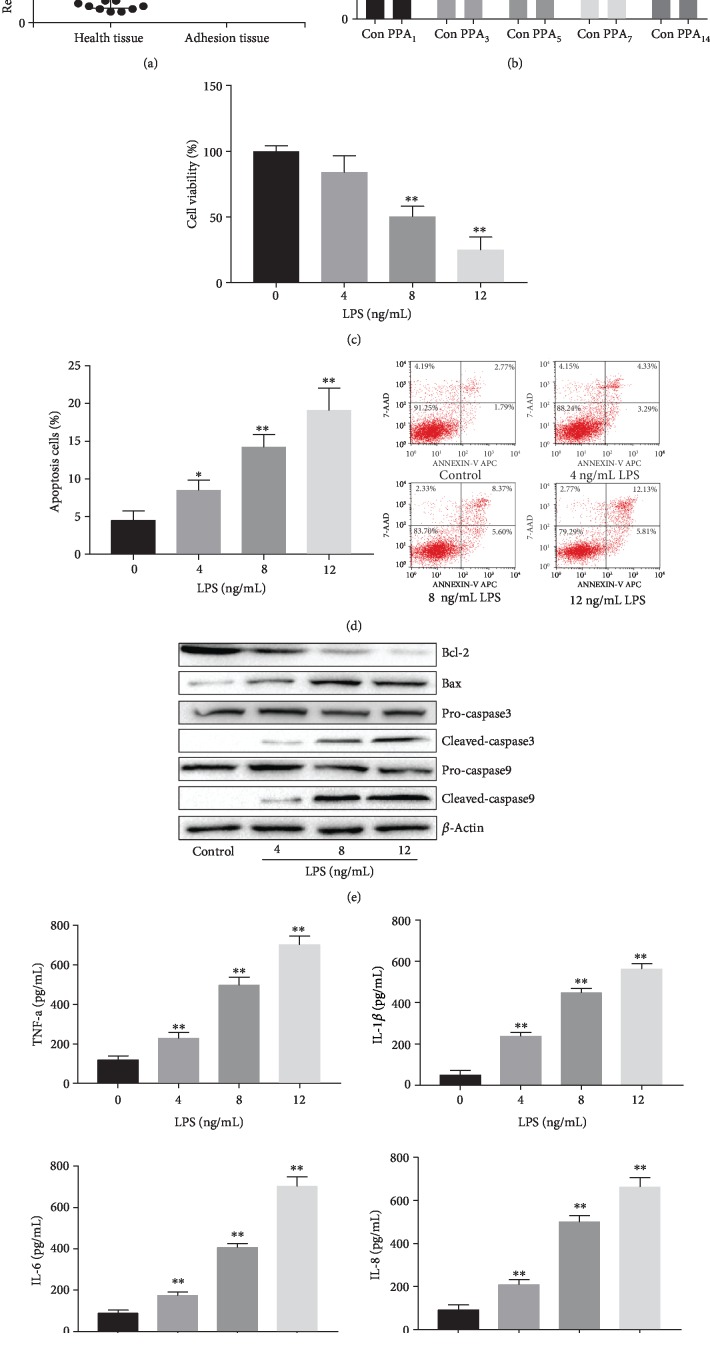
The high expression of Cox-2 was identified in adhesion tissues and LPS induced injury in HPMCs. (a) The expression of Cox-2 in adhesive tissues of patients with adhesive intestinal obstruction. (b) The expression levels of Cox-2 in rats adhesion tissues. (c) MTT assay of cell viability in HPMCs treated with different concentration of LPS (4, 8, 12 ng/mL). (d) Flow cytometry analysis of cell apoptosis in HPMCs treated with different concentration of LPS. (e) Western blot analysis of apoptotic-associated proteins in HPMCs after different LPS treatments. (f) ELISA analysis of the expression of inflammatory factors in supernatant of HPMCs after different LPS treatments. The experiments were repeated in triplicate. Significance: ^∗^*P* < 0.05 and ^∗∗^*P* < 0.01 versus the controls.

The expression levels of Cox-2 in human were detected among peritoneal adhesion samples from 16 patients who underwent laparotomy for adhesive intestinal obstruction and normal samples from 18 patients who underwent laparotomy for non-adhesive intestinal obstruction. We found that the expression of Cox-2 was highly expressed in adhesive groups compared with control groups (*P*<0.05, Figure 1(a)). In addition, the expression levels of Cox-2 in rat models were varied with the surgery time. When compared with the controls, respectively, there were significantly differences in PPA groups (*P*<0.05, Figure 1(b)). It suggested that Cox-2 might act as a key contributor in adhesion formation.

### 3.2. LPS Induced HPMCs Injury and Increased Cox-2 Expression

To determine the role of Cox-2 in HPMCs, HPMCs were treated with different concentration of LPS to prepare inflammatory model. It indicated that with the increase of LPS concentration, the cell viability was significantly inhibited and the cell apoptosis was markedly promoted (*P*<0.05, Figures 1(c) and 1(d)). We also found that with the increase of LPS concentration, the apoptosis-associated protein Bcl-2 was significantly decreased and Bax, Cleaved-caspase3 and Cleaved-caspase9 were markedly increased (*P*<0.05, Figure 1(e)). ELISA analysis suggested that the levels of inflammatory factors described above were dramatically increased (*P*<0.05, Figure 1(f)). In addition, we found that LPS with 8 ng/mL could reduce the viability of half present of HPMCs. We chose this concentration for the following research.

### 3.3. Effects of Cox-2 Suppression on Ameliorating LPS Induced HPMCs Injury

**Figure 2:**
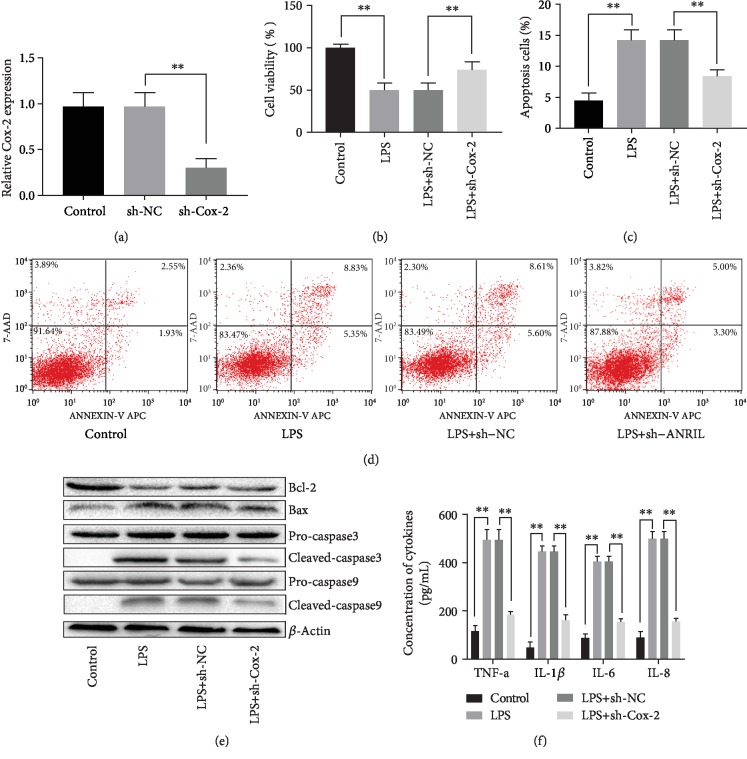
Effect of Cox-2 suppression on ameliorating LPS induced HPMCs injury. (a) qPCR analysis of the interference effect of sh-RNA on expression of Cox-2 in HPMCs. (b) Cox-2 suppression markedly reversed the reduced cell viability induced by LPS in HPMCs. (c) Cox-2 suppression markedly inhibited the cell apoptosis induced by LPS in HPMCs. (d) Cell apoptosis analyzed by Flow cytometry. (e) The expression of apoptosis-associated proteins after different transfections. (f) The concentration of inflammatory factors in supernatant of HPMCs after different transfections. The experiments were repeated in triplicate. Significance: ^∗^*P* < 0.05 and ^∗∗^*P* < 0.01 versus the controls.

To identify the biological function of Cox-2 in LPS induced HPMCs injury, HPMCs were transferred with sh-Cox-2 to make the suppression of Cox-2, and then the transfections were determined by RT-PCT. We found that Cox-2 significantly decreased in sh-Cox-2 group in comparison with sh-NC group, which suggested the successful transfection (*P*<0.05, Figure 2(a)). It also suggested that suppression of Cox-2 could ameliorate the LPS induced HPMCs injury. In briefly, compared with LPS + sh-NC group, the cell viability in LPS + sh-Cox-2 group was markedly promoted (*P*<0.05, Figure 2(b)), while the cell apoptosis in LPS + sh-Cox-2 group was significantly inhibited (*P*<0.05, Figures 2(c)-2(e)), and the concentration of inflammatory factors was decreased (*P*<0.05, Figure 2(f)).

### 3.4. Effects of Cox-2 Suppression on Ameliorating LPS Induced HPMCs Injury by Regulation of miR-21 Negatively

**Figure 3:**
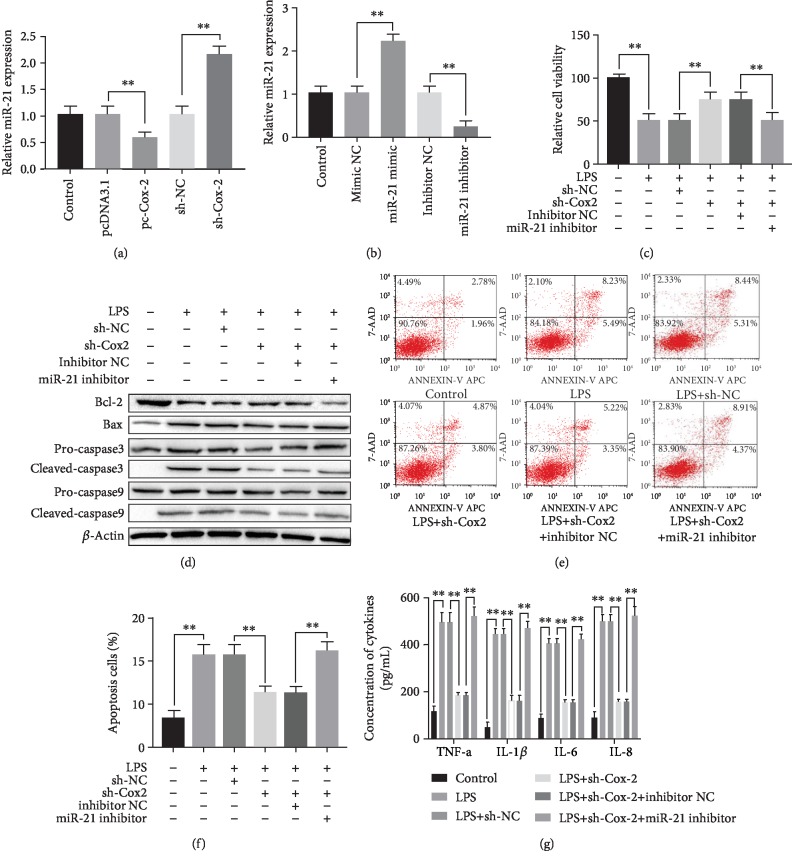
Suppression of Cox-2 ameliorating LPS induced HPMCs injury by regulation of miR-21 negatively. (a) Cox-2 negatively regulated the miR-21 expression after the HPMCs were transfected with pc-Cox-2, sh-Cox-2 and the corresponding controls. (b) The interference effect of miR-21 expression after the HPMCs were different transfected. (c) MTT assay showed cell viability after different treatments. (d) The expression of apoptosis-associated proteins after different transfections. (e) Cell apoptosis analyzed by Flow cytometry. (f) Flow cytometry showed cell apoptosis after different treatments. (g) The concentration of inflammatory factors in supernatant of HPMCs after different transfections. The experiments were repeated in triplicate. Significance: ^∗^*P* < 0.05, and ^∗∗^*P* < 0.01 versus the controls.

To understand whether the Cox-2 and miR-21 function as the competing endogenous RNAs (ceRNAs) on LPS induced cell injury. We performed the regulatory function between Cox-2 and miR-21. It indicated that the lower expressed miR-21 in pc-Cox-2 group relative to pcDNA 3.1 group and higher expressed in sh-Cox-2 group relative to sh-NC group. It suggested that Cox-2 might act as a negative regulator of miR-21 (*P*<0.05, Figure 3(a)). To further explore whether the exact role of Cox-2 on LPS induced HPMCs injury through regulating miR-21, the LPS-treated HPMCs were treated with miR-21 mimic and/or miR-21 inhibitor to make overexpressed and suppressed model. The transfection effect was determined by RT-PCR (*P*<0.05, Figure 3(b)). Subsequently, HPMCs were transfected with sh-Cox-2 and miR-21 inhibitor. The effect of cell viability and apoptosis, as well as the concentration of inflammatory factors were determined (*P*<0.05, Figures 3(c)-3(g)). The results showed that the function of Cox-2 on LPS induced HPMCs injury was achieved by regulation of miR-21 negatively.

### 3.5. miR-21 Negatively Correlated with TLR4, and TLR4 Was Targeted by miR-21

**Figure 4:**
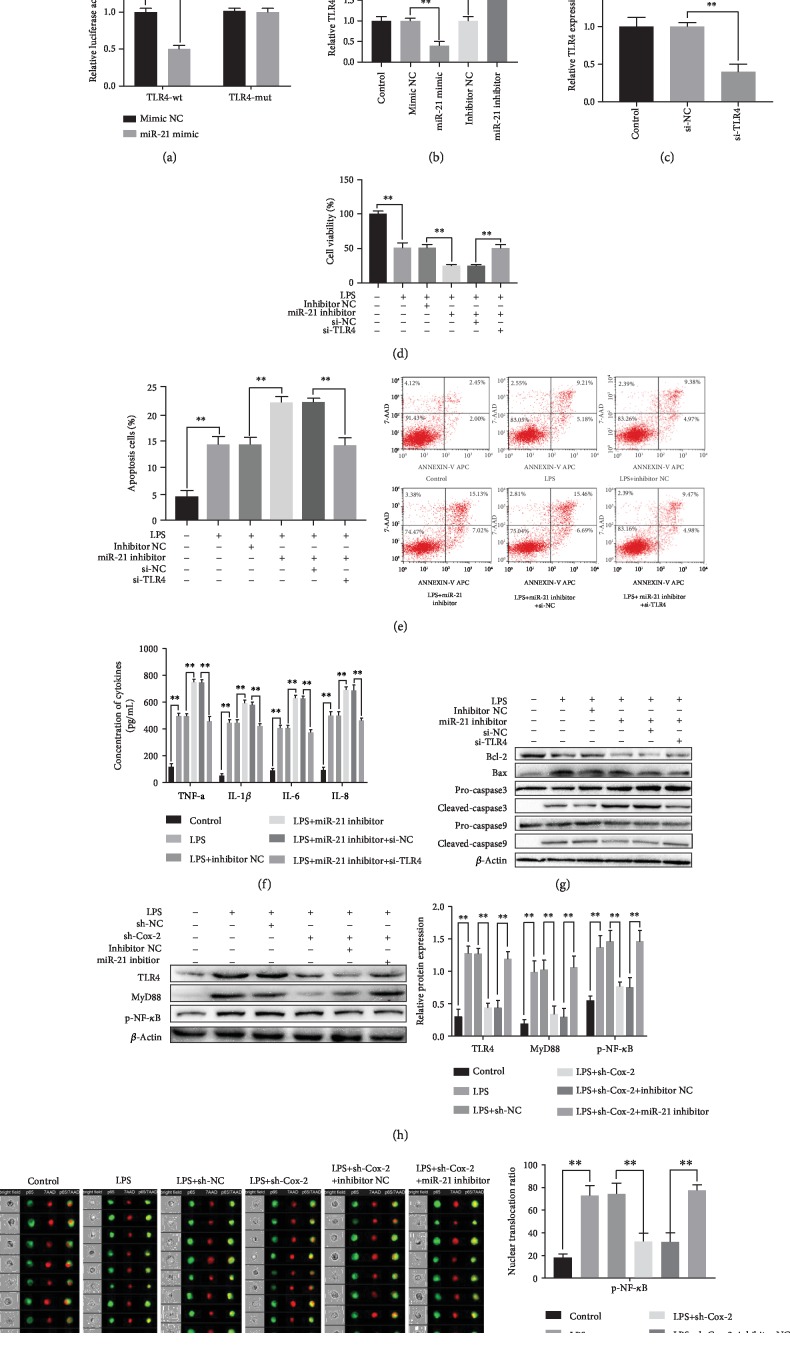
The negative regulation between miR-21and TLR4, and the regulatory mechanism of Cox-2 in LPS induced HPMCs injury via NF-*κ*B axis. (a) Target prediction of the binding sites between miR-21 and TLR4. Luciferase report of the luciferase activity between miR-21 and TLR4. (b) The expression of TLR4 after transfection with overexpressed and suppressive miR-21. (c) The TLR4 expression after transfection with si-RNAs. (d) MTT assay showed cell viability after different treatments. (e) Flow cytometry showed cell apoptosis after different treatments. (f) The concentration of inflammatory factors in supernatant of HPMCs after different transfections. (g) The expression of apoptosis-associated proteins after different transfections. (h) The expression of downstream genes of NF-*κ*B axis after different treatments. (i) NF-*κ*B nuclear translocation in HPMCs after different treatments by Flow cytometry. (j) The nuclear translocation ratio of NF-*κ*B. The experiments were repeated in triplicate. Significance: ^∗^*P* < 0.05 and ^∗∗^*P* < 0.01 versus the controls.

To explore the downstream contributors of miR-21, the relevant targets were predicted by using Targetscan online tool. In our study, TLR4 was identified as the potential target gene of miR-21. The blind sequence of both were presented in Figure 4(a). Then, we tried to verify whether the effect of miR-21 function was achieved by targeting TLR4, LPS-treated HPMCs were treated with miR-21 mimic and/or miR-21 inhibitor. We found that TLR4 was lower expressed in miR-21 overexpressed group and higher expressed in miR-21 suppressed group related to their control group (*P*<0.05, Figure 4(b)). It illustrated that the negative correlation among miR-21 and TLR4. Moreover, the relative luciferase activity of TLR4-wt was markedly inhibited in miR-21 mimic group in comparison with its control. However, there was no change in TLR4-mut group. It verified that TLR4 was directly targeted by miR-21.

### 3.6. Knockdown of TLR4 Ameliorated the Effects of miR-21 Suppression on LPS Induced HPMCs Injury

To further confirm the regulatory mechanism between miR-21 and TLR4. LPS-treated HPMCs were transfected with si-TLR4 and/or miR-21 inhibitors. In comparison with si-NC group, the expression of TLR4 was significantly decreased in si-TLR4 group, which suggesting the successful transfection (*P*<0.05, Figure 4(c)). In addition, we found that the cell viability were promoted and cell apoptosis were inhibited, as well as the concentration of inflammatory factors were decreased when knockdown of TLR4 (*P*<0.05, Figures 4(d)-4(g)). It suggested that the effects of miR-21 suppression on LPS induced HPMCs injury were ameliorated by knockdown of TLR4.

### 3.7. Effect of Cox-2 on LPS Induced HPMCs Injury via TLR4/MyD88/NF-*κ*B Signaling

TLR4 with its ligands MyD88, as well as their downstream signaling cascades, such as NF-*κ*B signaling was reported acting as potential pathway in inflammatory response and tissue injury [16]. In our study, we tried to explore the critical roles of TLR4/MyD88/NF-*κ*B signaling in LPS induced HPMCs injury. LPS-treated HPMCs were transfected with sh-Cox-2 and/or miR-21 inhibitor, and the expression levels of TLR4, MyD88 and NF-*κ*B were determined. We found that the expression of the above proteins were significantly decreased in sh-Cox-2 group, which indicated that knockdown of Cox-2 inhibited LPS induced activation of TLR4/MyD88/NF-*κ*B signaling. And we found that the protein expressions were further remarkably increased after miR-21 suppression (*P*<0.05, Figure 4(h)). Meanwhile, the nuclear translocation of NF-*κ*B p65 was significantly increased in the knockdown of both Cox-2 and miR-21 group (*P*<0.05, Figures 4(i)-4(j)).

## 4. Discussion

In the current study, we firstly tried to explore the biological functions among key lncRNAs, miRNAs, as well as the potential pathway involved in adhesion formation in molecular level. We found that lincRNA Cox-2 was highly expressed in peritoneal adhesion tissues compared with that in normal tissues both in human and rats. Then, HPMCs were treated with LPS to induce a vitro model of inflammatory injury. It indicated that the Cox-2 contributed toward LPS induced HPMCs injury. Suppression of Cox-2 reversed the cell viability and apoptosis, as well as the production of inflammatory factors in LPS induced HPMCs injury. Furthermore, Suppression of Cox-2 reversed the LPS induced HPMCs injury by regulation of miR-21 negatively. And we found that miR-21 was negatively correlated with TLR4, and TLR4 was predicted as target gene of miR-21. Furthermore, it showed that knockdown of TLR4 reversed the suppression of miR-21 on LPS induced HPMCs injury. Finally, we found that the effect of Cox-2 on LPS induced HPMCs injury was achieved by negatively regulation of miR-21 through TLR4/MyD88/NF-*κ*B signaling.

lincRNA Cox-2 is well studied in various kinds of biological process, such as migration and invasion of hepatocellular carcinoma [17], progression of liver fibrosis [18]. It is also suggested that Cox-2 function as an important regulator either in active or inactive expression of some immune genes, which further regulate inflammatory reaction [19]. Olfat G. Shaker et al. [20] reported that Cox-2 may serve as key biomarker in diagnosis of rheumatoid arthritis. Our results showed that Cox-2 was highly expressed in adhesion tissues. It was also supported by previous study [21]. Hence, we surmise that the Cox-2 plays a significant role in adhesion formation.

Mounting global evidences support that ceRNA act as essential functions in inflammatory pathway [22, 23]. However, studies on how the lncRNA and miRNA regulate inflammatory function in adhesion formation are still lack. In our study, we found that Cox-2 could negatively regulate miR-21, which was targeted with TLR4. Meanwhile, inhibition of miR-21 could reverse the suppression effect of Cox-2 on LPS induced HPMCs injury, which form a positive feedback loop. miR-21 is found in a variety of cancers [24], and some inflammatory disease [25, 26], as well as peritoneal fibrosis [27, 28]. Recent study [29] reported the role of miR-21 targeting of the TLR4/MyD88 in macrophages infected reaction. Our study also indicated that knockdown of TLR4 reversed the suppression of miR-21 on LPS induced HPMCs injury. Given the crucial player of TLR4 in inflammatory and immune pathway and taken the above evidence together, we argue that Cox-2 acts as a ceRNA by competing with miR-21 on its target TLR4 involved in the pathogenesis of inflammatory injury.

TLR4/MyD88/NF-*κ*B signaling pathway is a well-known pathway related to immune inflammatory responses and its activation is associated with multiple diseases, such as knee osteoarthritis [30], ulcerative colitis [31], and colorectal cancer [32]. However, there is no study report this signaling pathway on postoperative peritoneal adhesion. It was once suggested that TLR4/MyD88/NF-*κ*B signaling transduction may serve as a potential pathway for preventing peritoneal inflammation in peritoneal dialysis [33]. We assume that the NF-*κ*B axis may be the downstream regulatory pathway of LPS induced HPMCs injury. To shed light on this issue, we determined the expression of TLR4, MyD88 and NF-*κ*B in HPMCs transfected with sh-Cox-2 and/or miR-21 inhibitor after treated with LPS. We found that knockdown of Cox-2 inhibited the production of the downstream genes, which were further promoted after suppression of miR-21. And the result of NF-*κ*B p65 nuclear translocation expression was consistent with the results of Western blot. So we further speculate that Cox-2 plays a critical function on LPS induced injury by regulating NF-*κ*B p65 axis.

To sum up, our study suggest that the function role of lincRNA Cox-2 in LPS induced HPMCs injury. The suppression of Cox-2 could inhibit the inflammatory injury by negatively regulation of miR-21 and inactivation of TLR4/MyD88/NF-*κ*B signaling axis. These may provide new insights into preventing postoperative peritoneal adhesion.

## Data Availability

All data included in this study are available upon request by contact with the corresponding author. Correspondence should be addressed to Li Zeng; zengli@njucm.edu.cn.
